# Cervico-thoracic pain and associated impairments in air force personnel: a cross-sectional study

**DOI:** 10.1186/s12891-021-04301-w

**Published:** 2021-05-14

**Authors:** Matthias Tegern, Ulrika Aasa, Helena Larsson

**Affiliations:** 1 Department of Neurobiology, Care Sciences and Society, Division of Physiotherapy, Karolinska Institutet, Huddinge, Sweden; 2 Department of Community Medicine and Rehabilitation, Division of Physiotherapy, Umeå University, Umeå, Sweden; 3 Swedish Armed Forces, HQ, Stockholm, Sweden

**Keywords:** Fighter pilots, Helicopter pilots, Rear crew, Musculoskeletal disorders, Neck pain, Physical performance, Prevention, Range of motion, Movement control, Isometric strength and endurance

## Abstract

**Background:**

Pain and impaired function in the cervical region are common in Air Force personnel (AFP), but evidence is limited regarding the thoracic region. This cross-sectional cohort study examined associations between cervico-thoracic pain and physical performance among Swedish AFP and explored possible differences and similarities in test performance between fighter pilots (FP), helicopter pilots (HP) and rear crew (RC).

**Methods:**

AFP (*n* = 73) from one airbase performed eight tests of movement control of the spine, active cervical range of motion (ROM) in all six directions and isometric strength and endurance of the cervical flexors and extensors. The association between test performance and cervico-thoracic pain (based on the ‘Musculoskeletal screening protocol’ questionnaire) were analysed in a multiple binary logistic regression model.

**Results:**

For AFP with cervico-thoracic pain (30%), movement control was impaired in the ‘neck flexion test’ (OR [95%CI] =3.61 [1.06–12.34]) and the ‘forward lean test’ (OR [95%CI] =3.43[1.04–11.37]), together with reduced flexion ROM (OR [95%CI] =0.93 [0.87–0.99]). Test performance was in general similar between the three groups, but FP and HP could control the ‘forward lean test’ to a significantly higher degree than RC (*p* = 0.000). Further, FP showed significantly greater ROM in lateral flexion to the right compared to HP and RC (mean: 40.3°, 36.2° and 33.4°, respectively, *p* = 0.000), and they showed higher, although not significant, flexor strength than RC (*p* = 0.026).

**Conclusions:**

The impaired function associated with cervico-thoracic pain highlights the need for a deeper understanding of such relationships when designing tools to systematically optimize the physical performance and prevent pain among AFP. Studies with a longitudinal design are warranted to examine any causative associations between pain and impairments.

**Supplementary Information:**

The online version contains supplementary material available at 10.1186/s12891-021-04301-w.

## Introduction

The one-year prevalence of pain experienced by Swedish Air Force personnel (AFP) in the cervical and thoracic regions has been reported as 28 and 31% respectively [1]. The same study also found that this is more than two and half times higher than that reported by Swedish army soldiers (11 and 12% for each region, respectively) [1]. Internationally, AFP are also more prone to pain in the cervical region compared to non-flying military officers [2]. A recent systematic review and meta-analysis of fighter pilots (FP) found that neck pain prevalence was as high as 51% [3]. Prevalence for helicopter pilots (HP) has been reported from 43 to 67% [4–6] and 45 to 62% for helicopter rear crew (RC) [5, 7, 8]. Although neck pain is a well-known problem, a Dutch study indicated that the prevalence in fighter pilots is increasing [9]. Pain in the thoracic region has received less attention in the literature. One Austrian study did however report a prevalence of 28% for Austrian HP and 15% among RC [5]. Cervical and thoracic region pain is thus an occupational problem for the AFP community.

Work-related musculoskeletal disorders are likely of multifactorial origin [10] and the underlying mechanisms to neck pain are uncertain [11]. Physical factors suggested among AFP include increased muscular strain due to, in-flight adopted flexed postures and repeated movements that are biomechanically less favourable [12, 13], as well as wearing a helmet and helmet-mounted equipment [14]. These factors contribute to greater neck muscle load, as revealed by electromyographic activation levels [12–14]. Exposure to unfavourable postures and wearing flight-related equipment were also associated with pain among FP [15, 16], HP and RC [5]. In studies examining physical performance among AFP, poorer neck muscle strength, cervical range of motion (ROM), and motor control were more common among FP and HP with neck pain than among pain-free pilots [17–19].

A systematic review and meta-analysis showed that 39% of FP had lost time from flying due to neck pain [3]. The high prevalence of pain may have a pernicious effect on AFP physical condition and should thus be reduced to maintain flight performance, safety and operational readiness [20]. In Sweden, the Musculoskeletal Screening Protocol (MSP) [21] has already been implemented in the Army and will further be implemented in the Air Force. The MSP includes a questionnaire and tests of physical performance [21, 22], as well as individually tailored interventions based on the screening outcomes [23]. The physical performance test battery has so far included mainly neuromuscular tests for the torso and lower extremity [21, 24]. When selecting tests, it is important to assess factors associated with the experience of pain in the cervical and thoracic regions [10]. The present study therefore aimed to examine associations between pain in the cervical and thoracic regions, movement control, active cervical range of motion, and muscle strength and endurance in the same regions among Swedish AFP. A secondary aim was to compare test performance between FP, HP and RC.

## Methods

### Design and procedure

In this cross-sectional cohort study, Swedish AFP answered the MSP baseline questionnaire, performed eight tests of movement control of the spine [25], active cervical range of motion (ROM) in all six directions, and isometric strength and endurance in cervical flexion and extension in a standardized order (as presented under “physical performance testing”). One of the authors (HL) administered the questionnaires and assisted during the strength and endurance tests. The first author (MT), an experienced physical therapist (PT) who was blinded to the participants’ pain status performed the testing. For the strength and endurance tests, the PT asked participants about ongoing pain to determine whether the tests could be performed with regards to location and intensity. The Regional Ethical Review Board in Stockholm approved the study, DNR:2013/144–31/2 and DNR:2015/493–32.

### Participants

All AFP (only males) listed on flight duty at one airbase in Sweden during the period June 2015 to May 2016 were invited to participate in the study. Each AFP accepted the invitation and, following screening, all were included in the study (*n* = 73 [36 FP, 18 HP and 19 RC]).

### Questionnaire

The MSP questionnaire [1, 21–23, 26, 27] was used to gather information regarding age, height, weight, and one-year and point prevalence of musculoskeletal pain or injuries for ten body regions. For point prevalence, the AFP rated their maximal pain intensity from 0 to 10 using the numerical pain rating scale (NPRS). For the purpose of this study, only one-year and point prevalence in the cervical and thoracic regions were reported and combined as cervico-thoracic region pain. Questions regarding flight hours during the previous 12 months and total (i.e., career) flight hours were added.

### Physical performance testing

#### Movement control test battery

The tests included in the test battery (‘neck flexion in sitting’, ‘neck extension in sitting’, ‘neck rotation in sitting’ (left and right), ‘neck flexion in supine’, ‘chest lift’, ‘pelvic tilt’ and ‘forward lean’) are based on work by Sahrmann, and Comerford and Mottram [28–30]. All tests were performed in sitting with the feet in contact with the floor except for ‘neck flexion in supine’ that was performed lying on a bench with a small towel under the head, hands on the stomach and legs extended. The tests are used to analyse habitual movement patterns including relative flexibility [31] and/or challenge the ability to control movements in one region while moving an adjacent one [32]. All tests have been presented in detail previously and showed moderate to almost perfect inter-rater agreement (prevalence and bias adjusted kappa coefficients = 0.57–0.84) and fair to substantial test-retest agreement (prevalence and bias adjusted kappa coefficients = 0.33–0.69) [25]. Each test was evaluated with a dichotomous rating indicating whether the participant could [1] or could not (0) perform the movement according to the grading criteria (Appendix).

#### Active cervical ROM

**Fig. 1:**
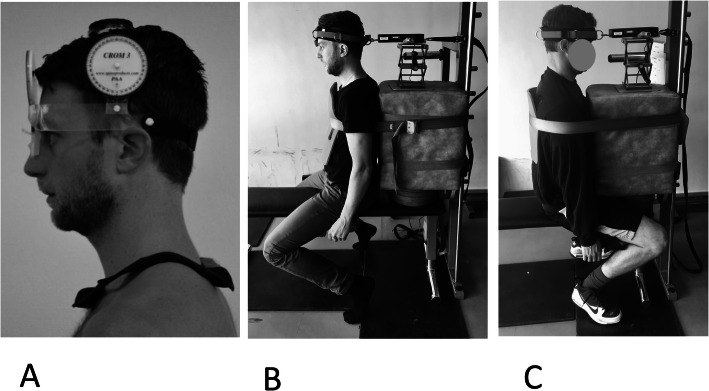
**a-c**. **a**: The CROM 3; **b** and **c**: Isometric flexor and extensor strength and endurance tests

The Cervical Range of Motion (CROM) 3 device (Performance Attainment Associates, Roseville, MN) was used to measure active cervical ROM (degrees, in the following order: flexion, extension, axial rotation left and right, and lateral flexion left and right) (Fig. 1 a). The CROM 3 has two gravity-controlled balls as well as a magnetic compass to measure movements in all three movement planes and has shown good reliability [33]. For testing, participants sat on a bench in a neutral upright position with hips and knees in 90°, hands resting on their thighs. To ensure full active ROM, they performed three repetitions of each movement. The highest value was used for analyses. To control movement in adjacent body regions during measurements of flexion and extension, the PT stood on the side of the bench with their hands on the participant’s sternum and thorax. To stabilize during measurements of rotation, the PT kneeled on the bench behind the participant with their hands on the participant’s shoulders. To stabilize during measurements of lateral flexion, the PT stood in front of the participants and held one hand on the opposite shoulder of the side being flexed.

#### Isometric strength and endurance in cervical flexion and extension

A fixed dynamometer (Advanced Force Gauge, Mecmesin Ltd., Slinfold, West Sussex, UK) measured the maximal voluntarily contraction (MVC) in cervical flexion and extension in an upright sitting position, similar to Lo Martire et al. [34] (Fig. 1 b-c). Participants warmed up using a rowing machine for 7–8 min in a self-selected pace, followed by five gradually increasing submaximal isometric contractions against the test leader’s hand in cervical flexion and extension. Participants wore a firm headband which was attached to a dynamometer positioned in line with the centre of the headband. To measure flexion, the back of the participants rested against a rigid square block, their knees were flexed so that only the tips of their toes were in contact with the floor, and a strap over the sternum fixated the arms and thorax. To ensure proper movement and prevent injury, participants kept their chin down (i.e., slight cranio-cervical flexion) to avoid protraction. Up to three submaximal isometric contractions were performed to ensure proper alignment and good position of the fixation and headband. Three trials were performed with a gradual onset of force to maximum for about three seconds with one-minute rests between trials. The gradual increase was intended to avoid injuries and falsely high values [18]. The average of the two highest measurements was used as the MVC and were multiplied with the lever arm (measured with a ruler as the vertical distance between the tragus of the ear and the spinous process of C7) to calculate the torque (Nm). After three minutes of rest, they were accustomed with the force needed to obtain 50% of their MVC. The assisting test leader kept the time and noted their perceived fatigue during the endurance test at 15 s intervals using the Borg CR10 scale [35]. The test leader monitored the force and gave verbal feedback to the participant if the force deviated 10 N from the intended torque. The test was interrupted if: (i) the participant was unable to maintain the correct torque after two encouragements by the test leader, (ii) a fatigue rating ≥ 7 on the Borg CR10 scale was reached, or (iii) any pain was experienced. To measure the MVC and endurance of the cervical extensors, participants sat on the bench with their sternum against the rigid square and the same procedure as for flexion was performed.

### Data handling and statistical analyses

Demographic data are presented as relative frequencies or means with standard deviations/min-max, or medians with interquartile range. Normal distribution of data was checked visually with q-q-plots, Shapiro-Wilk test and values of skewness and kurtosis. In cases of non-normal distribution, non-parametric tests were performed. Differences of age, height, weight, flight hours, active cervical ROM and isometric strength and endurance between FP, HP and RC were evaluated with analysis of variance (ANOVA) or Kruskal-Wallis test (with Bonferroni post-hoc adjustment). The one-year and point prevalence of cervico-thoracic pain, and performance of movement control tests were analyzed with Pearson Chi-square tests (with Bonferroni post-hoc adjustment when appropriate). Binary logistic regression analyses presented as odds ratios (OR) with 95% confidence intervals (CI) were performed to investigate the association between the dependent variable ‘cervico-thoracic pain’ and movement control, ROM, and isometric strength and endurance. Pain was defined as reporting any point prevalence of pain or injury in the cervical or thoracic region. First, univariate associations were checked, and factors associated with a *p*-value < 0.20 were deemed suitable to use in the multivariate regression model. Thereafter, factors associated with cervico-thoracic pain with a p-value > 0.05 were sequentially removed from the model to identify the model of best fit. Confounding, defined as a > 10% change in OR between the adjusted and crude model, was checked for a priori for possible confounders; age, group (i.e., FP, HP and RC) and pain in adjoining body regions (i.e., lumbar [25%]) and shoulder regions [11%]). No such change was evident and thus the crude model was presented. IBM SPSS Statistics for Windows, version 27 (IBM Corp., Armonk, N.Y., USA) was used to analyse the data. A *p*-value < 0.05 was considered statistically significant.

## Results

### Participant characteristics

Table 1 shows the characteristics of the included AFP. FP were significantly younger than HP ad RC (*p* = 0.000) while HP had logged significantly higher total flight time than FP and RC (*p* = 0.003).

**Table 1:** Demographic data of included participants; fighter pilots (*n* = 36), helicopter pilots (*n* = 18), rear crew (*n* = 19).

	All		FP		HP		RC		p-value
**Age (years)**	**39**	**(8)**	**35**^**a,b**^	**(7)**	**43**^**a**^	**(8)**	**43**^**b**^	**(7)**	0.000
Height (m)	1.81	(0.06)	1.82	(0.06)	1.80	(0.06)	1.78	(0.06)	0.078
Weight (kg)	82	(9)	81	(7)	85	(12)	79	(7)	0.144
TFT (h)	1500	(1625)	1400^a^	(1300)	2700^a,c^	(2125)	1000^c^	(900)	**0.003**
AFT (h)	110	(80)	120	(53)	113	(103)	68^d^	(94)	0.086

Data are presented as mean, SD with one-way ANOVA for age, height and weight, and median with interquartile range for TFT and ANFT.

TFT: Total flight time, AFT: annual flight time (i.e., previous 12 months), FP: Fighter pilots, HP: Helicopter pilots, RC: Rear crew.

*P*-values in bold represent significant difference between FP, HP and RC. Post-hoc analysis with Bonferroni adjusted *p*-values (*p* = 0.05/3): a: *p* < 0.017 between FP and HP. b: *p* < 0.017 between FP and RC. c: *p* < 0.017 between HP and RC. d: missing data, *n* = 3.

### Associations between cervico-thoracic pain and physical performance

The one-year/point prevalence for cervico-thoracic pain were 56/30% for all AFP combined and 58/22%, 50/39% and 58/36% for FP, HP and RC, respectively. The mean (min-max) NPRS rating for point prevalence was 4.5 (1.0-8.0) for all AFP and 5.0 (2.0–8.0), 3.9 (1.0–8.0) and 4.1 (1.0–6.0) for FP, HP and RC, respectively. There were no significant differences in pain prevalence between groups nor in demographic data in AFP with and without cervico-thoracic pain.

Table 2 shows the results from the physical performance tests. The univariate logistic regression identified three movement control and five ROM tests that were associated with cervico-thoracic pain (*p* < 0.20).

**Table 2:** Univariate logistic regression analyses for cervico-thoracic pain with physical performance tests (*n* = 73)

	Cervico-thoracic pain							
	Yes (*n* = 22)		No (*n* = 51)		Odds Ratio	95% CI		p-value
*Movement control* ^*a*^								
Neck flexion in sitting								
*Controlled*	23		55		1.0			**0.011**
*Uncontrolled*	77		45		4.14	1.32	12.94	
Neck extension in sitting								
*Controlled*	41		29		1.0			0.337
*Uncontrolled*	59		71		0.60	0.21	1.71	
Neck rotation left in sitting								
*Controlled*	41		55		1.0			0.273
*Uncontrolled*	59		45		1.76	0.64	4.84	
Neck rotation right in sitting								
*Controlled*	36		43		1.0			0.590
*Uncontrolled*	65		57		1.33	0.47	3.72	
Neck flexion in supine								
*Controlled*	73		59		1.0			0.259
*Uncontrolled*	27		41		0.54	0.18	1.60	
Chest lift								
*Controlled*	36		61		1.0			**0.055**
*Uncontrolled*	63		39		2.71	0.96	7.63	
Pelvic Tilt								
*Controlled*	50		55		1.0			0.700
*Uncontrolled*	50		45		1.22	0.45	3.31	
Forward lean								
*Controlled*	55		78		1.0			**0.039**
*Uncontrolled*	45		22		3.03	1.04	8.85	
*Range of motion (degrees)* ^*b*^								
Flexion	52.7	10.5	58.8	9.0	0.93	0.88	0.99	**0.015**
Extension	63.4	11.5	67.2	10.8	0.97	0.93	1.02	**0.186**
Rotation left	65.4	9.2	68.6	8.0	0.96	0.90	1.02	**0.146**
Rotation right	60.7	7.6	62.9	7.4	0.96	0.90	.1.03	0.252
Lateral flexion left	35.6	6.7	39.6	7.2	0.92	0.86	1.00	**0.030**
Lateral flexion right	36.0	5.5	38.1	6.7	0.95	0.87	1.03	**0.195**
*Isometric strength and endurance* ^*c*^								
Flexor (Nm)	25.0	16.8	27.6	8.7	0.98	0.90	1.07	0.621
Extensor (Nm)	40.1	11.9	39.8	9.7	.98	.89	1.07	0.658
Flexor (s)	54.0	27.0	54.0	21.0	1.00	.97	.1.04	0.884
Extensor (s)	106.0	64.0	92.0	44.0	1.00	.98	1.02	0.777

a: Data for movement control is presented in percentages

b: Data for range of motion is presented in mean (SD).

c: Data for strength and endurance are presented in median (interquartile range). Missing data for strength test and endurance; the cervico-thoracic pain group: ten and thirteen for flexor and extensor tests, respectively, for the no cervico-thoracic pain group: six participants for both flexor and extensor tests. Nm = Newton meters, s = seconds. P-values in bold indicates variables associated with cervico-thoracic pain with *p* < 0.20 and forwarded to multiple logistic regression model

The final multiple logistic regression model identified the movement control tests ‘neck flexion in sitting’ (OR, 95% CI: 3.61, 1.06–12.34, *p* = 0.040), ‘forward lean’ (OR, 95% CI: 3.43, 1.04–11.37, *p* = 0.044) and cervical flexion ROM (OR, 95% CI: 0.93, 0.87–0.99, *p* = 0.031) as significantly associated with cervico-thoracic pain (Table 3). Thus, less control of neck flexion and forward lean movement control tests, and lesser cervical flexion ROM were associated with cervico-thoracic pain.

**Table 3:** Multiple logistic regression analysis for cervico-thoracic pain with physical performance tests (*n* = 73)

	Initial model				Final model			
	Odds ratio	95% CI		p-value	Odds ratio	95% CI		p-value
*Movement control tests*								
Neck flexion in sitting								
Controlled	1.0			0.037	1.0			**0.040**
Uncontrolled	2.11	1.10	15.95		3.61	1.06	12.34	
Chest lift								
Controlled	1.0			0.238				
Uncontrolled	2.11	.61	7.24					
Forward lean								
Controlled	1.0			0.038	1.0			**0.044**
Uncontrolled	4.10	1.10	15.29		3.43	1.04	11.37	
*Range of motion (degrees)*								
Neck flexion	.92	.86	0.99	0.029	.93	.87	.99	**0.031**
Neck extension	1.05	.97	1.13	0.209				
Neck rotation left	0.98	.90	1.07	0.689				
Neck lateral flexion left	.90	.79	1.03	0.113				
Neck lateral flexion right	1.01	.89	1.15	0.843				

P-values in bold indicates a variable associated with cervico-thoracic pain with *p* < 0.05. Post-hoc analysis with cervical and thoracic region pain separately showed that for cervical region pain; cervical flexion ROM (OR, 95% CI: 0.92, 0.86–0.99, *p* = 0.019), and for thoracic region pain; the movement control tests; ‘neck flexion in sitting test’ (OR, 95% CI: 9.90, 1.15–85.04, *p* = 0.037) and ‘chest lift test’ (OR, 95% CI: 6.61, 1.17–36.6, *p* = 0.032) were significantly associated, respectively.)

### Comparisons of test performance between FP, HP and RC

The results for movement control, ROM and isometric strength and endurance tests are presented in Table 4. For movement control, the groups differed significantly only for the forward lean test (Pearson Chi-square 15.2, *p* = 0.000), with a post-hoc analysis revealing that fewer RC could control the movement compared to both FP and HP. For ROM, a significant difference was found only for lateral flexion to the right (*p* = 0.000), with a post-hoc analysis revealing that FP had higher ROM compared to HP and RC. For strength, a difference was found for flexors (*p* = 0.026), for which the post-hoc analysis revealed greater strength for FP than RC. This difference was, however, not significant after Bonferroni adjustment.

**Table 4:** Results of physical performance tests for fighter pilots (FP), helicopter pilots (HP) and rear crew (RC) (*n* = 73)

	FP		HP		RC		
*Movement control* ^*a*^	(*n* = 36)		(*n* = 18)		(*n* = 19)		p-value
Neck flexion in sitting							
*Controlled*	53		33		42		0.381
*Uncontrolled*	47		67		58		
Neck extension in sitting							
*Controlled*	42		28		21		0.262
*Uncontrolled*	58		72		79		
Neck rotation left in sitting							
*Controlled*	58		61		26		0.046
*Uncontrolled*	42		39		74		
Neck rotation right in sitting							
*Controlled*	50		39		26		0.231
*Uncontrolled*	50		61		74		
Neck flexion in supine							
*Controlled*	26		12		8		0.083
*Uncontrolled*	10		6		11		
Chest lift							
*Controlled*	56		56		53		0.828
*Uncontrolled*	44		44		47		
Pelvic tilt							
*Controlled*	64		44		42		0.207
*Uncontrolled*	36		56		58		
Forward lean							
*Controlled*	86^d^		78^e^		37^d, e^		**0.000**
*Uncontrolled*	14		22		63		
*Range of motion (degrees)* ^*b*^							
Flexion	56.7	8.6	55.4	10.6	58.8	11.2	0.564
Extension	68.8	11.7	63.0	11.5	63.7	13.1	0.113
Rotation left	67.8	9.2	69.7	9.4	65.3	8.7	0.292
Rotation right	63.9	7.7	62.0	7.9	59.3	7.9	0.089
Lateral flexion left	40.2	7.5	38.4	5.0	34.7	7.6	0.027
Lateral flexion right	40.3^d, f^	6.1	36.2^f^	5.1	33.4^d^	5.8	**0.000**
*Isometric strength and endurance* ^*c*^							
Flexor (Nm)	28.6	10.2	24.9	6.3	24.4	8.7	0.026
Extensor (Nm)	41.8	11.6	41.7	10.6	36.8	3.4	0.091
Flexor (s)	54.0	19.0	50.0	26.0	58.0	21.0	0.688
Extensor (s)	92.3	36.4	100.4	33.0	120.6	36.8	0.065

a: Data for movement control is presented in percentages with the Chi-square test

b: Data for range of motion is presented in mean (SD) with one-way ANOVA.

c: Data for strength and endurance are presented in median (interquartile range) with Kruskal-Wallis test. P-values in bold represent significant difference between FP, HP and RC with Bonferroni adjustment (*p* = 0.05/3). Post-hoc analysis: *d* = *p* < 0.017 between FP and RC. *e* = *p* < 0.017 between HP and RC. *f* = *p* < 0.017 between FP and HP. Missing data for strength and endurance; FP: *n* = 3 and *n* = 5 for flexor and extensor tests, respectively; HP: *n* = 5 and *n* = 6 for flexor and extensor tests, respectively; RC: *n* = 8 for both flexor and extensor tests. Nm = Newton meters, s = seconds

## Discussion

The main findings of this study were that an impaired ability to control flexion movements in the cervical (OR = 3.61) and lumbar (OR = 3.43) regions, as well as less cervical flexion ROM (OR = 0.93), were significantly associated with self-reported cervico-thoracic pain among Swedish AFP.

### Movement control

The observation that AFP with cervico-thoracic pain performed neck flexion with an uneven distribution of motion along the cervical spine and diminished anterior sagittal plane rotation (‘neck flexion in sitting test’, OR = 3.61) is in line with earlier studies where altered movement coordination strategies were shown for cervical flexors in Swedish HP with pain [17]. To be able to perform a smooth neck flexion with the load of the head evenly distributed along the cervical spine, it is necessary to have sufficient mobility in all cervical segments and to include the deep cervical flexors in the flexion muscle synergy [36]. The electromyography study by Ang (2008) showed a higher activity in the superficial sternocleidomastoid muscle in HP with neck pain compared to pain-free colleagues, thus indirectly showing a lower activity in the deep cervical flexors during the cranio-cervical flexion test [17] as previously presented by Falla et al. [37]. A greater activity in superficial muscles that are not able to perform smooth movements has also been shown in patients with mechanical neck pain [38].

FP are exposed to extremely high acceleration gravitational forces (i.e., G-load) and perform rapid head movements sometimes during g-loading [15], whereas HP and RC are exposed to long-lasting static positions [12, 13]. The weight of their helmets and, during darkness, also mounted visual aiding systems (e.g., night vision goggles) induce even more extensive head movements [9]. The strength and endurance of the superficial muscles among AFP must therefore be sufficient to cope with such loads. Well-functioning muscle coordination strategies are additionally important in order to evenly distribute the load of the head, helmet and helmet-mounted equipment. Among AFP with cervico-thoracic pain, it seems that neck flexion is characterized by insufficient deep cervical flexors activity in the flexion muscle synergy as visually observed during the ‘neck flexion test’. Further, the ‘forward lean test’ assesses the ability to flex the hips and lean forward to about 30° without lumbar flexion. The finding that the lumbar spine moves more readily than the hips for AFP with cervico-thoracic pain (forward lean, OR = 3.43), is likely related to the biomechanically less advantageous sitting postures in the cockpit which are associated with the shape of the back rest and safety vest worn [9].

Most movement control tests were, however, not associated with experiencing cervico-thoracic pain. A possible reason is the large heterogeneity in the neuromuscular adaptations accompanying pain disorders [39] and the fact that our tests had a dichotomous scale. A more comprehensive scoring system to grade sensorimotor control tests has been suggested [40].

### Range of motion

Flexion and lateral flexion to the left was univariately significantly associated with cervico-thoracic pain, although only flexion remained in the final multiple model. A lesser ROM in AFP with cervico-thoracic pain compared with those without is in agreement with earlier studies in FP [19] and HP [17] which showed both lesser sagittal (flexion-extension) and transversal plane (bilateral rotation) ROM. Interestingly, Van den Oord et al. (2010) found no difference between HP and RC with neck pain compared to pain-free peers [41]. Comparisons between studies is however complicated because studies from different countries use different measurement methods. Impairments in ROM seem nevertheless to be an important physical feature associated with pain in this region. An important consequence of this is that AFP require good ROM to maintain an adequate field of view, but if ROM is restricted in one region, then this movement is likely compensated for by an adjoining region which can subsequently experience pain [31].

### Strength and endurance

No significant differences between participants with and without cervico-thoracic pain were found in strength and endurance of cervical flexors and extensors. This finding is in line with studies including FP [19, 42] and HP [18, 41]. Ang et al. conversely reported lower extensor strength in FP with neck pain compared to their pain-free colleagues [18]. The contradicting strength results may be due to differing devices used across studies and also that unlike earlier studies, we excluded AFP who reported pain that may have been worsened by the test. Apart from strength measures, De Loose et al. (2009:2) found, however, significant differences in neck muscle morphology using magnetic resonance tomography [42]. Namely, the semispinalis and multifidus muscles but not superficial muscles (including the trapezius, levator scapulae or splenius muscles) showed about 1/3 greater cross-sectional area in FP reporting neck pain in the last 12 months compared to pain-free FP. Further, these muscles showed significantly greater cross-sectional area on the left side compared to the right side in FP with pain but not in pain-free. The literature on these morphological differences is, however, conflicting [42].

### Differences and similarities between fighter pilots, helicopter pilots and rear crew

The different working environments and tasks of FP, HP and RC expose each to contrasting physical loads. The total flight hours were significantly higher for HP compared to both FP and RC. This can partially be explained by that occasionally FP convert to HP later in their career thus continuing to fly for more years in another air system. This in turn is correlated to a significantly higher age of HP compared to FP. However, the annual flight time did not significantly differ between groups. The impaired flexion movement control of RC in their lumbar region (i.e., the forward lean test) may be due to sitting and standing for prolonged periods with a bent or twisted torso [8]. The higher ROM for FP in right lateral flexion compared with both HP and RC was accompanied by trends of higher values for left lateral flexion, extension and right rotation. The FP were also close to significantly stronger than RC in the flexion test. These findings are not surprising considering that FP need to move their cervical spine to extreme positions of combined extension, rotation and lateral flexion under high G-forces to maintain visual contact with their enemy [15]. The fact that FP were also significantly younger might have contributed to these findings since cervical ROM decreases with higher age [43]. Although the number of AFP in this study was not sufficient for further sub-group comparisons, preventive and clinical attention may still need to be specific to FP, HP and RC personnel [18].

Certain methodological considerations related to our study should be noted to facilitate future research. The cross-sectional study design allowed for investigation of associations between physical test performance and experience of pain, which is important during developmental of the MSP for AFP [10, 44]. The definition of pain and body regions in this study was equivalent to earlier Swedish studies including air force and army personnel [1, 21–23, 26, 27] in which the participants answer if they have had any physical complaints or injuries during the previous 12 months and/or at present. Other definitions of pain, such as work- or flight-related, were not included because this would have made comparisons of study results challenging [3]. We also combined pain in the cervical and thoracic regions as they often accompany each other [1, 5]. Thoracic pain and dysfunction is also common in many cervical disorders [45]. The movement control tests in this study have shown adequate (moderate to almost perfect) inter-rater agreement but lower (fair to substantial) test-retest agreement, which should be considered in follow-up situations [25]. Different systems for measuring cervical ROM and cervico-thoracic strength exist and could affect results. Regarding endurance testing for example, the study by Alricsson et al. [46] used a fixed value of 196 N held for as long as possible, whereas we used 50% of MVC for both flexors and extensors. One disadvantage of a relative over a fixed value is the uncertainty that the participant has performed their true maximal effort during MVC testing. Finally, to avoid aggravating pain among those with existing symptoms, not all participants performed isometric strength and endurance tests and thus due to missing data the related results should be interpreted with caution.

### Strengths and limitations

To our knowledge, this is the first study to investigate associations between pain and physical performance in the cervico-thoracic region using three cohorts of Swedish AFP. The included tests were time-efficient and easy to apply and can therefore easily be integrated in regular testing of AFP. Further, we included all available AFP at one airbase; our findings are therefore mainly generalizable to Swedish AFP on duty, although the findings may also be relevant for AFP of other countries, and other groups of workers with high loads on the upper spine. Although we only included physical features associated with cervico-thoracic pain, we are aware that the individual’s pain experience must always be considered from a biopsychosocial perspective. We have shown in a previous study that lower self-reported rating of physical health was associated with increased odds of upper body pain in AFP [1]. The MSP questionnaire covers both work- and leisure-time factors, together with psychological and social domains that have shown to be important to reduce the burden of musculoskeletal disorders in the Swedish army. Other measures, including general fitness, are likely also of importance for the origin of cervico-thoracic pain [47]. Lastly, we included all AFP listed on flight duty at one Swedish airbase and no power calculation was performed. This might contribute to the fact that few differences in physical performance between FP, HP and RC were found. Still, their different physical work exposure suggests that AFP-specific preventive tools/screening tests should be taken into consideration.

## Conclusions

This study showed that impaired movement control of the cervical and lumbar regions, as well as cervical range of motion, were associated with cervico-thoracic pain in Swedish AFP. Therefore, assessment of physical performance including movement control and ROM seems an appropriate addition to the existing MSP to systematically optimize physical performance and prevent pain among AFP. Strength and endurance in the cervico-thoracic region were not associated factors and therefore seem to be less important for cervico-thoracic pain on a group level, although this needs to be investigated further. Prospective studies are also required to investigate potential causative associations between physical test performance and cervico-thoracic pain in AFP. We further suggest that future studies include a more detailed assessment of AFP with cervico-thoracic pain to fully establish their individual impairments.

## Supplementary Information

**Additional file 1.**

## Data Availability

There are ethical restrictions regarding data availability for public release in this study since identification of participants from the data cannot be ruled out. Data contained in this paper are considered as sensitive. According to the Ethical committee in Sweden, and within the Swedish Armed Forces, we are not allowed to have data available for public release due to ethical restrictions. We can only make the data available upon reasonable request, which will also involve discussions with the Swedish Armed Forces. Contact information: Swedish Armed Forces. Research coordinator. Anders Claréus. 107 85 Stockholm/Sweden. Phone: + 46 8 788 85 26. E-mail: anders.clareus@mil.se

## Abbreviations

AFP: air force personnel
AFT: Annual flight time
ANOVA: analysis of variance
CI: confidence intervals
FP: fighter pilots
HP: helicopter pilots
MSD: musculoskeletal disorders
MSP: musculoskeletal screening protocol
MVC: maximal voluntarily contraction
NPRS: numerical pain rating scale
OR: odds ratio
RC: rear crew
ROM: Range of motion
SD: standard deviation
TFT: Total flight time
