# Supplementary material for: Cervico-thoracic pain and associated impairments in air force personnel: a cross-sectional study
Source: BMC Musculoskelet Disord. 2021 May 14;22:441. doi: 10.1186/s12891-021-04301-w (PMC8122543; doi:10.1186/s12891-021-04301-w)
Supplement: Supplementary file 1 — Additional file 1. [file 12891_2021_4301_MOESM1_ESM.pdf]

Appendix to: Cervico-thoracic pain and associated impairments in air force personnel: a cross-sectional cohort study.

Matthias Tegern, Ulrika Aasa and Helena Larsson

Movement control tests; instructions and grading criteria.

Prior to each test, a short video of the test was shown to the participants together with verbal instructions. The participants repeated the movement three times to ensure familiarization with the movement to be tested, thereafter they performed the movement and the PTs rated whether the participant could (1) or could not (0) perform the movement according to the grading criteria (i.e. “pass” or “fail” of the test). The tests have been presented earlier in textbooks (1,2). In the case a participant could not perform the test according to grading criteria, the reason was noted (see below). No feedback regarding test outcome was given during or after the test. The order of the tests was maintained throughout the study beginning with tests in sitting followed by supine. The tests are here presented according to body regions.

Neck flexion in sitting test

Adapted from (1)

The purpose was to assess the ability to flex the neck to 45°–50° with contribution of both lower (≈35°) and upper cervical spine without cervical anterior translation/ diminished anterior sagittal plane rotation.

PABAK[a] inter-rater: 0.78 (0.58-0.98), test-retest: 0.38 (0.11-0.65).

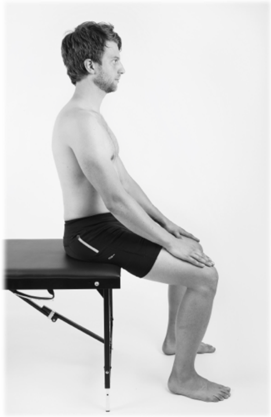

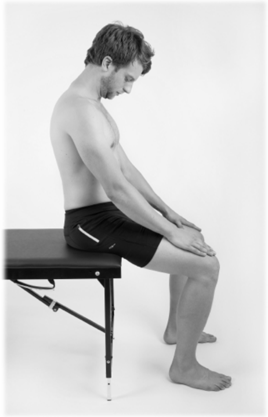

Participants were sitting on a bench with the feet on the floor, hands on the thighs and the neck, and back and shoulders in a neutral position. From this position, participants were instructed to flex their neck as far as possible, and then to return to the start position. The participants were instructed not to move the upper body during the movement.

Grading criteria: The test was evaluated as passed if the lower and upper cervical spine was contributing to flexion concurrently to 45°–50° (visually, whereof about 35° was performed in the lower cervical spine). It was evaluated as failed if there was increased or decreased flexion in the lower cervical spine, if there was increased or decreased flexion in the upper cervical spine, or if there was an anterior translation (head forward) of the head and cervical spine with diminished anterior sagittal plane rotation during neck flexion.

a: PABAK: Prevalence and bias adjusted kappa coefficients (with 95% confidence intervals).

Published in: Tegern M, Aasa U, Äng BO, Harms-Ringdahl K, Larsson H (2018) Inter-rater and test-retest reliability of movement control tests for the neck, shoulder, thoracic, lumbar, and hip regions in military personnel. PLOS ONE 13(9): e0204552.

Neck extension in sitting test
Adapted from(1)

The purpose was to assess the ability to extend the neck to ≈85° with contribution of both lower (≈70°) and upper cervical spine without mid-cervical anterior translation.

PABAK[a] inter-rater: 0.62 (0.37-0.87), test-retest: 0.56 (0.31-0.80).

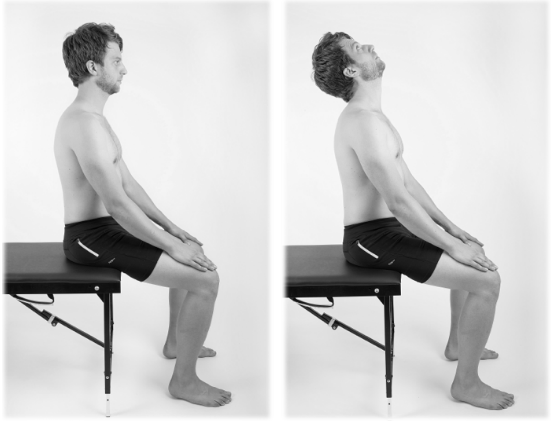

Participants were sitting on a bench with the feet on the floor, hands on the thighs and the neck, and the back and shoulders in a neutral position. From this position, participants were instructed to extend the neck as far as possible to look up at the ceiling, and then to return to the start position. The participants were instructed not to move the upper body or the shoulders during the movement and to keep the mouth closed.

Grading criteria: The test was evaluated as passed if the lower and upper cervical spine was contributing to extension concurrently to about 85° (visually, whereof about 70° was performed in the lower cervical spine). It was evaluated as failed if there was increased or decreased extension in the lower cervical spine, if there was increased or decreased extension in the upper cervical spine, or if there was a mid-cervical anterior translation (hinge) during neck extension.

Neck rotation test (left and right)
Adapted from (2)

The purpose was to assess the ability to rotate the neck to about 70°–80° without concurrent neck or shoulder movements.

Left/right: PABAK[a] inter-rater: 0.78 (0.58-0.98)/0.68 (0.44-0.91), test-retest: 0.33 (0.06-0.61)/0.64 (0.42-0.87).

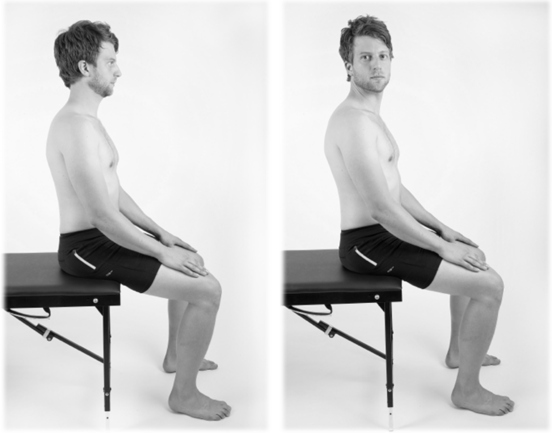

Participants were sitting on a bench with the feet on the floor, hands on the thighs and the neck, and the back and shoulders in a neutral position. From this position, participants were instructed to turn the head to the side as far as possible without bending the neck, and then to return to the start position. The participants were instructed not to move the upper body or the shoulders during the movement.

Grading criteria: The test was evaluated as passed if there was a cervical rotation to 70°–80° with no concurrent flexion (eyes kept horizontal) or extension or any shoulder movements. It was evaluated as failed if there were flexion, or lateral flexion of the neck or an excessive or early rotation in the thoracic spine or movements of the scapula during neck rotation.

a: PABAK: Prevalence and bias adjusted kappa coefficients (with 95% confidence intervals).

Published in: Tegern M, Aasa U, Äng BO, Harms-Ringdahl K, Larsson H (2018) Inter-rater and test-retest reliability of movement control tests for the neck, shoulder, thoracic, lumbar, and hip regions in military personnel. PLOS ONE 13(9): e0204552.

Neck flexion in supine test

Adapted from (1)

The purpose was to assess the ability to smoothly flex the neck using all cervical segments without excessive anterior translation.

PABAK[a] inter-rater: 0.84 (0.66-1.0), test-retest: 0.47 (0.21-0.72).

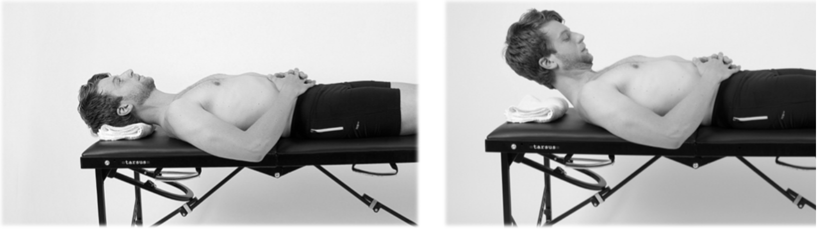

Participants were in a supine position on a bench, their arms lying on their belly with their head resting on a small towel to allow the neck to be in a neutral position. From this position, participants were instructed to flex their upper cervical spine by lightly holding the chin in followed by lifting the head off the bench without lifting the back, and then to return to the start position with a maintained upper cervical flexion.

Grading criteria: The test was evaluated as passed if the participant was able to smoothly flex their neck using all cervical segments, and then return to start position. The test was evaluated as failed if there was an excessive anterior translation in relation to the amount of anterior sagittal rotation or if the movement was jerky, indicating impaired muscle-recruitment pattern between intrinsic and extrinsic neck flexors.

Chest lift test

Adapted from (2)

The purpose was to assess the ability to extend the thoracic spine (lifting the chest) without anterior pelvic tilt and lumbar extension.

PABAK[a] inter-rater: 0.57 (0.30-0.83) test-retest: 0.60 (0.37-0.83).

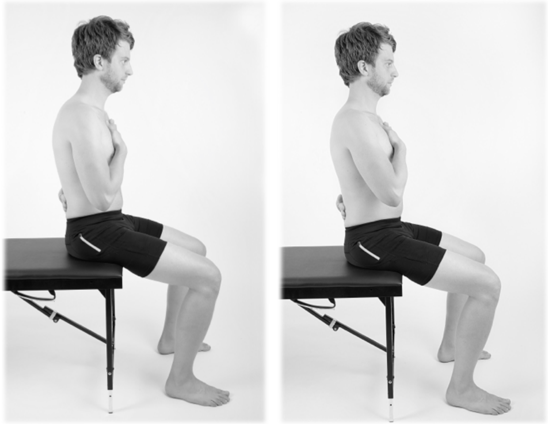

Participants were sitting on a bench with the feet on the floor, hands on the thighs and the neck, and low back and shoulders in a neutral position. The thoracic region was in slight flexion. From this position, participants were instructed to extend the thoracic region thus moving the sternum up and forward while keeping the lumbar region neutral, and then to return to the start position.

Grading criteria: The test was evaluated as passed if there was a thoracic extension without lumbo-pelvic movements. It was evaluated as failed if there was anterior pelvic tilt and lumbar extension during thoracic extension.

a: PABAK: Prevalence and bias adjusted kappa coefficients (with 95% confidence intervals).

Published in: Tegern M, Aasa U, Äng BO, Harms-Ringdahl K, Larsson H (2018) Inter-rater and test-retest reliability of movement control tests for the neck, shoulder, thoracic, lumbar, and hip regions in military personnel. PLOS ONE 13(9): e0204552.

Pelvic tilt test
Adapted from (2)

The purpose was to assess the ability to tilt the pelvis posteriorly without thoracic flexion.

PABAK[a] inter-rater: 0.78 (0.58-0.98) test-retest: 0.69 (0.48-0.90).

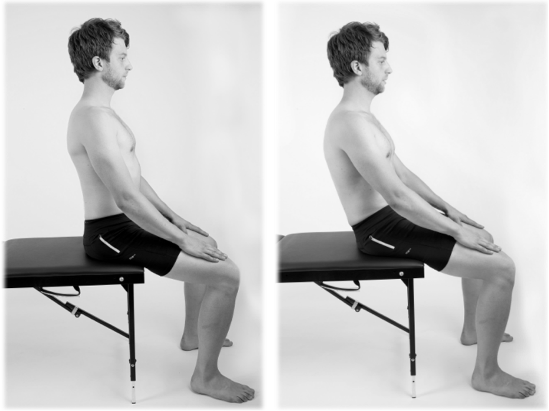

Participants were sitting on a bench with the feet on the floor, hands on the thighs and the neck, and the back and shoulders in a neutral position. From this position, participants were instructed to actively roll the pelvis backwards into posterior pelvic tilt while keeping the thoracic region neutral, and then to return to the start position.

Grading criteria: The test was evaluated as passed if there was a posterior pelvic tilt without thoracic flexion. It was evaluated as failed if there was a thoracic flexion during pelvic tilt.

Forward lean test
Adapted from (2)

The purpose was to assess the ability to flex the hip/lean forward to about 30° without lumbar flexion.

PABAK[a] inter-rater: 0.68 (0.44-0.91, test-retest: 0.42 (0.15-0.69).

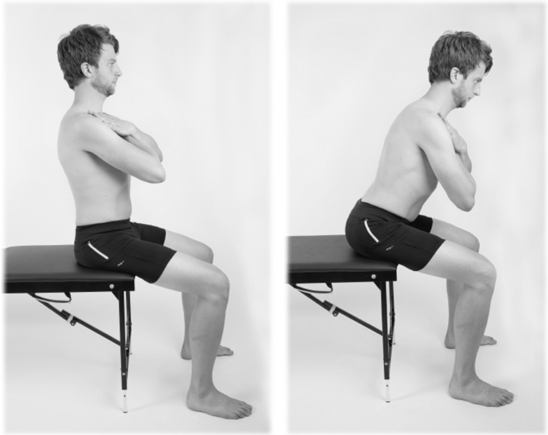

Participants were sitting on a bench with the feet on the floor, arms crossed on the chest and the neck, and the back and shoulders in a neutral position. From this position participants were instructed to flex their hips thus leaning their upper body forward to about 30° of flexion while keeping the lumbar region neutral, and then to return to the start position.

Grading criteria: The test was evaluated as passed if there was a hip flexion without lumbar flexion. It was evaluated as failed if there was a lumbar flexion or active extension during hip flexion.

a: PABAK: Prevalence and bias adjusted kappa coefficients (with 95% confidence intervals).

References:

1. Sahrmann S. Movement system impairment syndromes of the extremities, cervical and thoracic spines. St. Louis, Mo: Mosby; 2011.
2. Comerford M. Kinetic control : the management of uncontrolled movement. In: Mottram S, editor. Rev. ed. ed. Chatswood, N.S.W.: Chatswood, N.S.W. : Elsevier Australia; 2012.

Published in: Tegern M, Aasa U, Äng BO, Harms-Ringdahl K, Larsson H (2018) Inter-rater and test-retest reliability of movement control tests for the neck, shoulder, thoracic, lumbar, and hip regions in military personnel. PLOS ONE 13(9): e0204552.
